# Prometheus unbound? Oceanic affectivity and the neuropsychodynamics of addiction

**DOI:** 10.3389/fpsyg.2026.1699816

**Published:** 2026-02-17

**Authors:** Human-Friedrich Unterrainer

**Affiliations:** 1 Faculty of Psychotherapy Science, Sigmund Freud Private University, Vienna, Austria; 2 Institute for Religious Studies, University of Vienna, Vienna, Austria; 3 ARH - Addiction Research Hub, Grüner Kreis Ltd., Vienna, Austria; 4 University Department of Psychiatry and Psychotherapeutic Medicine, Medical University of Graz, Graz, Austria

**Keywords:** addiction, altered states of consciousness, default mode network, ego dissolution, existential neuroscience, neuropsychodynamics, oceanic affectivity, symbolization

## Abstract

Altered states of consciousness range from psychotic disintegration to mystical union, with oceanic affectivity - experiences of ego dissolution, boundlessness, and affective merging - constituting a central feature. While such states can be induced by psychedelics, they also occur through trauma, meditative practices, or within compulsive addictive cycles. In addition, oceanic states may offer temporary relief from psychic pain but often reinforce a regressive return to early undifferentiated states of self, bypassing affect and symbolic integration and adaptive regulation. From a neuropsychodynamic perspective, this dynamic reflects both a defense against and a longing for psychic wholeness. In that sense, addiction may function as a failed form of transcendence - an attempt to escape internal fragmentation through altered consciousness. Drawing on the myth of Prometheus, it is proposed that the repeated self-harming cycle of addiction resembles the eternal devouring of the liver: a neurobiological and symbolic site of both detoxification and self-destruction. Neuroscientifically, such states are associated with alterations in the default mode network, limbic dysregulation, reduced top-down control, and increased neural entropy. These changes may loosen rigid ego structures and facilitate the emergence of unconscious material, including early affective and relational imprints. This paper integrates psychodynamic theory with current neuroscientific findings to conceptualize addiction not merely as a maladaptive behavior but as a neuropsychodynamic attempt to restore lost internal cohesion. Understanding oceanic states as transitional phenomena at the boundary between breakdown and transformation offers a more nuanced approach to the addictive process and its therapeutic implications.

## 1 Introduction

I gave a gift to mortal beings. I searched out and stole the source of fire concealed in fennel stalks[…]. Now chained and nailed beneath the open sky, I am paying the price for what I did.—Aeschylus, Prometheus Bound, lines 107–113.

Oceanic affectivity, first articulated by Romain Rolland and discussed by Sigmund Freud, describes a profound sense of boundlessness, a feeling of oneness with the external world that predates the emergence of a differentiated self. Freud theorized this as a remnant of the infant’s undifferentiated ego relationship with the maternal object, suggesting that adult experiences of dissolution or unity may reflect regression to that primitive state (Ackerman, 2017; Freud, 1930: Saarinen, 2014). In more recent psychodynamic terms, such oceanic states reveal both the allure of fusion and the dangers of ego collapse or dissolution (Kainer, 2013; Sleight et al., 2023). Within addictive pathology, these affective experiences emerge paradoxically as both escape and entrapment. Therefore, addiction may be read as a dysregulated pursuit of oceanic states - an attempt to bypass psychic fragmentation through affective fusion, yet trapped in compulsive repetition and disintegration (Henden et al., 2013; Khantzian, 1997). Hereby, the myth of Prometheus, eternally bound and subjected to the daily violation of his liver, serves as a powerful metaphor: the liver - linked both symbolically and physiologically to renewal and toxicity - mirrors the addict’s internal cycles of craving and self-destruction (Winship, 1999).

Neuroscientific research supports this psychodynamic framing. Recent neuroimaging findings suggest that states of oceanic boundlessness, particularly during psilocybin administration, are marked by functional hyperconnectivity and a loosening of anti-correlated network dynamics (Mortaheb et al., 2024). From a neuropsychodynamic perspective, such unconstrained connectivity reflects a temporary suspension of inhibitory control, a transient state that may support affect integration or regression, depending on the availability of affective containment (Gergely and Watson, 1996; Solbakken et al., 2012; Zachrisson and Wong, 2023). Dysfunctional patterns in the Default Mode Network (DMN) - such as heightened posterior DMN connectivity and weakened anterior DMN engagement - have been consistently documented in substance use disorders and related behavioral addictions, correlating with impaired self-monitoring, craving, and relapse vulnerability (Koob and Volkow, 2016; Zhou et al., 2007). Additional findings implicate disrupted cross-network dynamics among the DMN, salience, and executive control networks, undermining cognitive regulation and emotional integration (Fuchshuber et al., 2025a; Sutherland et al., 2012). These patterns parallel oceanic affectivity wherein top-down control gives way to elevated neural entropy and susceptibility to unconscious affective material (Carhart-Harris et al., 2014; Tagliazucchi et al., 2016). By synthesizing psychodynamic concepts with network neuroscience, this paper proposes that addiction can be conceptualized as a neuropsychodynamic impasse, where oceanic longing, affective regression, and brain dysconnectivity coalesce. Exploring this transitional space offers transformative insights into the nature of addictive mentalities and their treatment.

## 2 Prometheus and the addictive mind: a neuropsychodynamic view

**Figure 1:**
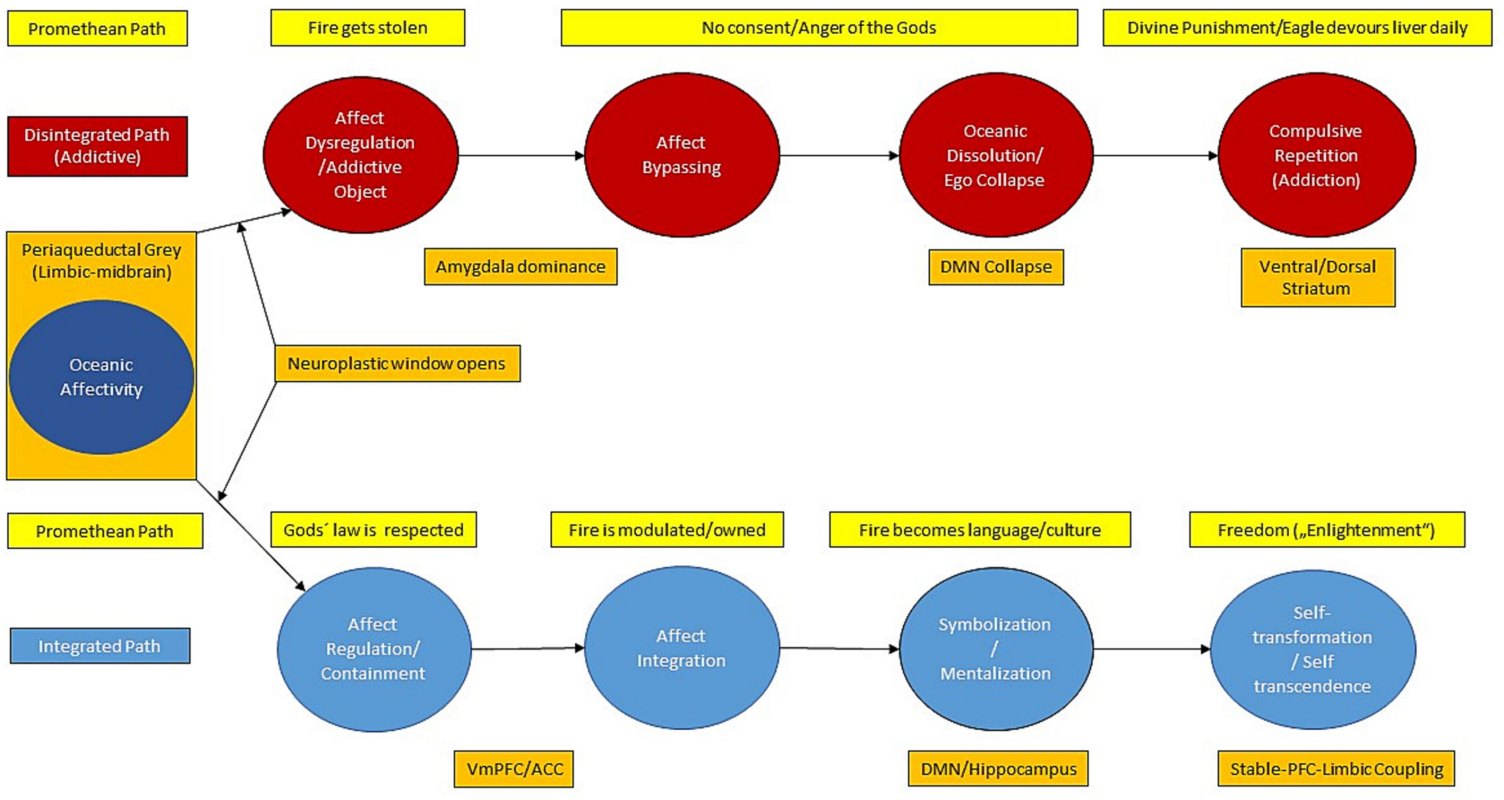
The way of Prometheus: a mytho-neuropsychodynamic model of affective transformation in addictive disorders. Mythological (yellow), neurobiological (orange), and psychodynamic (red/blue) interpretations of Prometheus’ two diverging paths (red, disintegrated; blue, integrated) caused by high oceanic affectivity. VmPFC/ACC, ventromedial prefrontal cortex/anterior cingulate cortex; DMN, default mode network; Oceanic affectivity, mythologically coded as “Promethean fire,” corresponds neurobiologically to the activation of limbic-midbrain structures (Alcaro et al., 2017) and characterizes a window of increased neural plasticity. From this threshold onwards, affective processing divides into two paths: when affects are regulated and contained (“in the sense of the gods”), they can be integrated and symbolized through reflective self-processing, supported by the coupling of the prefrontal and limbic cortex and the activation of the default mode network, leading to lasting self-transformation. When affects are dysregulated and circumvented (“the gods are betrayed”), symbolization fails, leading to oceanic dissolution, ego collapse, and the activation of reward- and habit-based neural systems that stabilize compulsive repetitions and cumulative physiological costs (mythologically encoded as daily consumption of the liver). These divergent outcomes are mythologically encoded as “enlightenment” versus “divine punishment.”

The myth of Prometheus, who defied the gods by stealing fire and suffered eternal punishment, has long served as a metaphor of transgression, suffering, and the cost of consciousness. Bound to a rock, his liver - organ of regeneration and purification - is devoured daily by an eagle, only to regrow each night. In the context of addiction, this myth resonates powerfully with the cycle of craving, intoxication, guilt, and collapse, followed by temporary repair and repetition (Winship, 1999). The liver, both in symbolic and biological terms, becomes the site of a repeating trauma - a physical and emotional wound that never fully heals. From a neuropsychodynamic perspective, this cycle reflects a deep split in the addicted psyche - between the drive toward transcendence and the unintegrated affective residues of early trauma (Alcaro et al., 2021; Fuchshuber and Unterrainer, 2020; Unterrainer, 2025a). Here, addiction can be understood as a dysfunctional attempt to restore psychic equilibrium through a chemically or behaviorally induced state of oceanic fusion - akin to the “fire” Prometheus steals (Khantzian, 1997; Winship, 1999). However, instead of illumination, what follows is fragmentation, remorse, and somatic degradation. This loop mirrors what Winnicott (1965) described as the collapse of the transitional space: the inability to symbolically process affect leads to either dissociation or compulsive action in addictive diseases (see Figure 1; also Renner et al., 2025 for further discussion).

Neurobiologically, these dynamics are not merely metaphorical. The repeated pursuit of euphoric or oceanic states is associated with maladaptive changes in brain networks involved in self-regulation and affect processing. The default mode network (DMN), particularly the medial prefrontal cortex and posterior cingulate cortex, plays a key role in self-referential thinking and autobiographical memory (Buckner et al., 2008; Carhart-Harris et al., 2014; Unterrainer, 2025b). In addition, hyperactivity in certain DMN regions correlates with ruminative craving and impaired reality testing (Zhou et al., 2007). Impaired top-down regulation from the executive control network may undermine the capacity for affective processing, increasing the likelihood of concrete enactments such as substance use, bingeing, or compulsive repetition. Such difficulties in integration often have roots in early developmental experiences (Music, 2014). Within this framework, the addictive cycle may transiently release psychic tension by loosening top-down control mechanisms that normally suppress unconscious affective material (Mary et al., 2020). At the same time, experiences of ego-dissolution can reduce the meta-cognitive conflict between urge and resistance (Millière, 2017), offering momentary relief yet reinforcing repetition when underlying conflicts remain unintegrated.

In both classical and contemporary psychoanalysis, the capacity to regulate affect and inner states is shaped by early relational experiences. When the caregiving environment fails to adequately mirror, regulate, or mentalize an infant’s distress, the developing psyche may lack a stable inner container for emotional experience (Fonagy and Target, 2002). In such cases, addiction can later emerge as an artificial regulator - a neurochemical or behavioral prosthesis that temporarily mimics the soothing presence of the early caregiver (Gergely and Watson, 1996; Khantzian, 1997). Consistent with Bowlby’s attachment theory (1969/1982), addiction can be conceptualized as an attachment disorder rooted in early relational disruptions that compromise secure affect regulation (Khantzian, 1997). Consequently, substance use often serves as a maladaptive substitute for the comfort and regulation typically provided by secure attachment figures (Flores, 2001; Hiebler-Ragger and Unterrainer, 2019; Schindler, 2019). In line with this assumption, Unterrainer et al. (2017) examined neural correlates of attachment insecurity in individuals with poly-drug use disorder (PUD). Using Diffusion Tensor Imaging, we identified impaired white matter integrity, particularly in the superior longitudinal fasciculus and superior corona radiate, among PUD patients compared to recreational drug users and non-users. These white matter abnormalities correlated with more insecure attachment styles and elevated negative affectivity, reinforcing the conceptualization of addiction as an attachment disorder at both psycho-behavioral and neurobiological levels (Flores, 2001; Fuchshuber et al., 2025b). Although constrained by its cross-sectional design, this study highlights the potential value of addressing attachment patterns in addiction treatment and underscores the critical role of brain-behavior interactions in substance use disorders (see also Unterrainer, 2025c for a more extensive overview).

The Promethean metaphor thus reflects both the existential drive for more - more feeling, more connection, more fire - and the price paid when the psyche lacks the capacity to regulate and integrate that intensity (Flores Mosri, 2021;). Neuroscientifically, this can be viewed as a form of increased neural entropy without integration (Carhart-Harris et al., 2014), where altered connectivity patterns lead to temporary dissolution of ego boundaries, but without the structural supports required for symbolization and self-transformation (see also Figure 1). In psychedelic research, similar oceanic experiences - often marked by ego dissolution and increased global connectivity, can facilitate therapeutic breakthroughs when integrated into a supportive relational context (Griffiths et al., 2016; Roseman et al., 2018).

In contrast, addiction often replicates these states without containment, leaving the individual suspended in unprocessed affect and somatic dysregulation (see Unterrainer, 2025b for further discussion). The fire is stolen, but there is no adequate space in which to carry it home. The neuropsychodynamic perspective emphasizes this failure of transformation. The addictive act does not represent mere pleasure-seeking, but an attempt - often unconscious - to return to a fused, undifferentiated state where pain is abolished and wholeness is temporarily restored (Flores Mosri, 2021; Unterrainer, 2025a). This echoes Freud’s notion of the “death drive” (Freud, 1920; see also Kirsch et al., 2022), not as a literal wish for death, but as a pull toward inorganicity, toward the cessation of psychic tension. Prometheus’s repeated punishment thus becomes a metaphor for this oscillation between transcendence and collapse (Unterrainer, 2025a). Therefore, to understand addiction fully, we must read it not only through the lens of neurobiology or behavioral conditioning but also as a mythic and affective process: one that enacts unresolved conflicts between self and other, mind and body, longing and containment. The task of treatment, then, is not simply to extinguish the fire, but to help the patient develop the appropriate tools to carry it - without being consumed (see Unterrainer, 2025c for further discussion).

## 3 Oceanic states as transitional phenomena: between regression and transformation

Oceanic affectivity occupies a paradoxical position in both clinical and neuroscientific discourse (Unterrainer, 2025b). On one hand, sensu S. Freud it is associated with regression to early undifferentiated states, potentially signaling the breakdown of ego boundaries and affective capacities. On the other, it may mark a threshold experience - a transient state with transformative potential when safely held and integrated [see also Schmautz et al., 2024 and Straßnig et al., 2025 for initial steps of an operationalization of this concept]. In the context of addiction, this paradox becomes especially pronounced: the pursuit of oceanic dissolution can offer temporary relief from psychic pain while simultaneously deepening structural disorganization (Unterrainer, 2025a). As already said, in psychoanalytic theory, oceanic states are often linked to primary narcissism and early symbiotic fusion with the maternal object (Kristeva, 1989; Mahler et al., 1968). Correspondingly, when reactivated in adulthood - whether through trauma, substance use, or ecstatic experiences - these states may overwhelm the ego’s integrative functions. Yet, they also contain the seeds of psychic renewal. As Bion (1965) and also Winnicott (1965) emphasized, the capacity to tolerate unstructured experience without disintegration is foundational to psychological growth. In that sense, oceanic affects may serve as both a regressive pull and a developmental challenge.

Neuroscientifically, these threshold-like states correspond to transient increases in neural entropy, functional disintegration of the default mode network, and altered patterns of global connectivity - features observed in both psychedelic states and certain addictive episodes (Carhart-Harris et al., 2014; Tagliazucchi et al., 2016). During such episodes, ego-related processing may be suspended, allowing previously inaccessible affective and autobiographical material to surface. When supported by a reflective container (e.g., therapeutic setting, ritual structure), this can lead to reorganization and integration. Without such containment, however - as in compulsive substance use - the experience may remain unmentalized, reinforcing psychic fragmentation. Prometheus remains chained to the rock and his liver is devoured by the eagle on a daily basis (see, e.g., Schimmenti et al., 2025 for further discussion; Winship, 1999).

Addiction reflects a disturbed in-between state, where overwhelming affect is repeatedly experienced but never properly integrated. Instead of symbolization, the addict repeats. The Promethean motif recurs here with renewed relevance. The fire that Prometheus brings – a metaphor that stands for illumination, creativity, and transcendent knowledge is also a dangerous force (Fink et al., 2014). The addict, like Prometheus, reaches for the fire of affective intensity, but without the inner maturity required to bear it. The punishment is not only divine but structural: the liver, a site of bodily regulation, is injured again and again in the absence of affective integration (Mehu, 2015; Winship, 1999). This interplay between regression and transformation invites a reframing of oceanic states within clinical neuroscience (Unterrainer, 2025b).

Rather than pathologizing them outright, these states can be understood as in-between spaces - transient thresholds between order and chaos, identity and dissolution, trauma and healing (Bromberg and Schore, 2012). Within such spaces, the self is destabilized but not yet reconstituted; the eventual outcome - whether psychotic breakdown or psychic integration - depends less on the experience itself than on the surrounding context and the subject’s developmental history (Schore and Schore, 2014).

From a clinical perspective, acknowledging the oceanic dimension of addictive experience may open new avenues of intervention. Rather than focusing narrowly on symptom control or behavioral abstinence, treatment might instead aim to support the patient’s capacity to mentalize unbearable affective states (Bateman and Fonagy, 2012). In certain cases, this may involve creating conditions in which oceanic states can be re-experienced under safe and containing circumstances - through relational depth, guided imagery, or even controlled psychedelic-assisted therapy (Carhart-Harris and Friston, 2019; Griffiths et al., 2016; Grof, 1985; Yu et al., 2024). Moreover, ritual and therapeutic framing not only protect against ego-collapse but also guard against ego-inflation that may accompany uncontained oceanic or ecstatic states. As Tzu and Damgaard (2014) observe, when boundaryless affect is not adequately contained, addictive behaviors can themselves become narcissistic enactments - forms of self-reinforcement that imitate transformation while deepening psychic fixation.

Such approaches do not seek to abolish transient states but to restore the affective and symbolic capacities required to process them (Kristeva, 1989). Ultimately, the challenge of addiction is not only neurobiological or psychodynamic, but also a mythologically inspired one: it concerns the failure to carry fire without being consumed by it (Winship, 1999). Reframed in this way, oceanic affectivity appears not merely as a clinical threat but as a threshold of transformation (see Figure 1).

## 4 Implications and conclusion: toward an symbolic integration of oceanic states

The neuropsychodynamic understanding of addiction challenges reductionist models that isolate neural mechanisms from subjective experience. It proposes instead a multidimensional view in which neurobiology, unconscious processes, and symbolic meaning converge (Unterrainer, 2025c). Oceanic affectivity exemplifies this convergence. Once viewed solely as mystical or regressive (Freud, 1930), it is now recognized as a key affective experience in states of ego dissolution - whether triggered by trauma, psychedelics, or compulsive substance use (Carhart-Harris et al., 2014; Letheby and Gerrans, 2017; Loizzo, 2012).

In addition, oceanic longing can be understood as a drive toward affective totality in the face of inner fragmentation. This longing reflects early failures in affect mirroring, symbolization, and the capacity to mentalize distress (Fonagy and Target, 2002; Gergely and Watson, 1996). When these developmental functions are compromised, the subject may resort to compulsive enactment - reaching again and again for the fire of affective release, yet unable to symbolize the experience. This might be seen as the Promethean dilemma: “Craving for transcendence”, but being bound to repetition (Unterrainer et al., 2013; see also Unterrainer, 2025b for further discussion). The repeated injury to the liver - a site of both literal and a digestion - becomes a metaphor for failed integration. Recent neuroscientific work underscores this cycle. Substance use disorders are marked by altered activity in the default mode network (DMN), particularly in medial prefrontal and posterior cingulate regions - areas implicated in self-referential thinking and autobiographical memory (Buckner et al., 2008; Sutherland et al., 2012; Zhou et al., 2007). Increased connectivity in posterior DMN regions has been linked to craving and rumination, while anterior hypoactivity reflects impaired self-monitoring. Disruptions in salience and executive control networks further destabilize the balance between introspection and regulatory control (Menon, 2011; Zilverstand et al., 2018). These findings align with the entropic brain hypothesis, which posits that elevated neural entropy (i.e., decreased functional differentiation among networks) underlies states of ego dissolution - whether in psychedelics, psychosis, or compulsive states like addiction (Carhart-Harris et al., 2014; Tagliazucchi et al., 2016). In such states, top-down control loosens, allowing unconscious affective material to surface. If this material can be mentalized, the result may be insight. If not, it risks psychic disorganization and compulsive repetition (Allen et al., 2008; Bromberg and Schore, 2012). This suggests that addiction is not simply a disease of “too much dopamine” or “too little willpower”, but a neuropsychodynamic impasse: an attempt to regulate unmentalized affect through compulsive repetition (Khantzian, 1997). The role of therapy, then, is not only behavioral management but psychodynamic restoration. Relationally attuned interventions, including mentalization-based therapy (Bateman and Fonagy, 2012), affect-regulation psychotherapy (Schore and Schore, 2014), and psychodynamic approaches that work with regression (Gabbard, 2005), all point to the centrality of containment and symbolization (Zachrisson and Wong, 2023). Even psychedelic-assisted therapy, when structured and relationally grounded, has shown potential for catalyzing integration of oceanic or ego-dissolving states (Griffiths et al., 2016; Roseman et al., 2018).

In sum, addiction reflects the psyche’s desperate attempt to resolve a fundamental failure of affect integration and symbolization (Cheetham et al., 2010; Solbakken et al., 2012; Sweet, 2012). Here, oceanic affectivity is not the problem nor the solution in itself - it is a signpost, pointing to deeper unmet needs for containment, coherence, and meaning. By linking neural entropy to psychic entropy, and regression to unrealized transformation, the neuropsychodynamic model offers a powerful lens for understanding and treating the addictive mind (see also Unterrainer, 2025a, 2025b, 2025c). Within this framework, the fire Prometheus steals can be read as the unregulated affective and cognitive energy characteristic of intense altered states. Empirical mapping of such states highlights their ambivalent qualities—simultaneously ecstatic, illuminating, and destabilizing (see Prugger et al., 2022 for further discussion). Whether this “fire” becomes transformative or destructive depends on the individual’s capacity for affective and symbolic containment and the presence of a supportive relational context (Kemp, 2018). Prometheus’s fire - traumatic, ecstatic, unregulated - must not be extinguished, but carefully carried forward. And that requires a holding environment: neurobiologically stable, relationally attuned, and rich in meaning.

## Data Availability

The original contributions presented in the study are included in the article/supplementary material, further inquiries can be directed to the corresponding author.
